# Life Insurance and Diseases of the Ear

**Published:** 1907-07-27

**Authors:** 


### Otology.

### LIFE INSURANCE AND DISEASES OF THE EAR.

Insurance companies are becoming more alive to the risks they run in accepting the insurance of individuals with certain diseases of the ear. It may be well to consider the following classes of disease.

1. Those diseases of the ear which involve no additional risk to life.

2. Those diseases in which there is a small additional risk to be considered.

3. Those in which the risk is greater, but might be conditionally accepted at a higher additional premium.

4. Those diseases which would justify absolute rejection of a candidate for life insurance.

5. Cases in which acceptance should be postponed for reconsideration at a later period.

1. The diseases which involve no additional risk to life.

Malformations and deformities or other affections without deafness, discharge or vertigo, can be regarded by life insurance officers as unimportant, and not influencing the duration of life of the individual. The same may be said of eczema without stenosis, furunculosis without middle-ear disease or systemic infection, and all affections of the middle-ear which occur without suppuration and in which the disturbance to hearing is not great. Also all middle-ear suppurations which have completely recovered and with the tympanic membrane soundly healed.

2. The diseases in which a small additional risk is to be considered.

The deaf individual runs risks greater than one who is not deaf. In a young subject, therefore, if very deaf, it would be fair to charge an additional premium, say, by adding five, six, or seven years to his age; while in later life, under similar circumstances, two or three years' addition to the age would be reasonable for this defect when unassociated with other manifestations of disease.

### Increased Premiums.

3. The diseases in which the risk, though greater, might be conditionally accepted at a higher additional premium.

A perforation in the tympanic membrane, even when there is no discharge, is associated with a greater risk of recurrence than in those cases in which the membrane is completely cicatrised. It may not be out of place to add that a membrane which has really healed sometimes appears as if perforated, owing to the presence of a thin concave scar.

The existence of vertigo as a rule precludes acceptance of a candidate for insurance. If accepted the rates should certainly be increased, not only on account of the risk brought on by the uncertainty of equilibrium, but also on account of other grave conditions which the existence of the vertigo may first call to notice.

4. Cases in which the "life" should be unconditionally rejected.

A medical adviser to an insurance company is justified in rejecting individuals with (1) tuberculous ulceration of the pinna or external canal; (2) atresia associated with otorrhœa; (3) otorrhœa with polypi or granulations or facial paresis or palsy, or with mastoid fistula; (4) otorrhœa with occasional pyrexia or with giddiness or tinnitus or vertigo or headache; (5) all cases of malignant disease.

5. Cases in which acceptance should be deferred, but may be reconsidered at a later period, as a rule with increased premiums.

### Doubtful Cases.

The following may be deferred until completely cured, and may then be accepted;—(1) All syphilitic diseases of the ear, the risks being as for syphilis; (2) all cases of eczema with diffuse swelling of the meatal walls, preventing examination of the tympanum, should be deferred until cured; (3) those cases of chronic suppurative middle-ear disease in which otorrhœa is the outstanding feature, and in which no threatening complication has ever existed. It has been suggested that if, after treatment, the otorrhœa cease, and the patient has no recurrence, his proposal may be accepted after two years, with an additional premium of five years. Whereas after a completely successful radical operation with the cavity everywhere free to inspection, and found to be soundly healed, and remaining so for over a year, the "life" may be accepted without additional premium.

In the case of intracranial complications, no proposal should be considered for quite ten years, but may certainly be accepted then, provided no cause for rejection on any other count is found.

The cases which carry most risk and are likely to be considered most frequently, are those of chronic suppurating otitis media. In private practice middle-ear suppuration is met with in nearly 10 per cent of aural cases. In hospital practice the percentage of middle-ear suppuration is certainly higher, for the lower classes are careless of the more trivial affections and neglect to properly carry out treatment for chronic suppuration.

Of hospital patients with aural affections 7 per cent. are admitted to the wards. Among these there is a mortality of nearly 20 per cent., showing the serious conditions for which they are admitted. While this relatively high mortality is to be partly accounted for by the class of patient ignorant of the results of neglect, it should be recognised that though suppurative middle-ear disease is a condition which is curable by treatment, it is one also which endangers life so long as it lasts. Exactly to what extent this danger exists there is no consensus of opinion, but exist it does, even when the only manifestation is an occasional and intermittent otorrhœa.

The figures are based upon 15,000 aural cases in private and hospital practice.

